# Supplementary material for: The interactome of intact mitochondria by cross-linking mass spectrometry provides evidence for coexisting respiratory supercomplexes
Source: Mol Cell Proteomics. 2017 Dec 8;17(2):216–32. doi: 10.1074/mcp.RA117.000470 (PMC5795388; doi:10.1074/mcp.RA117.000470)
Supplement: Supplemental Data [file supp_RA117.000470_133922_0_supp_23978_fzffwf.zip › spectra_annotation/mito_DR_spectra_annotation/88-1-10-1-2-1.pdf]

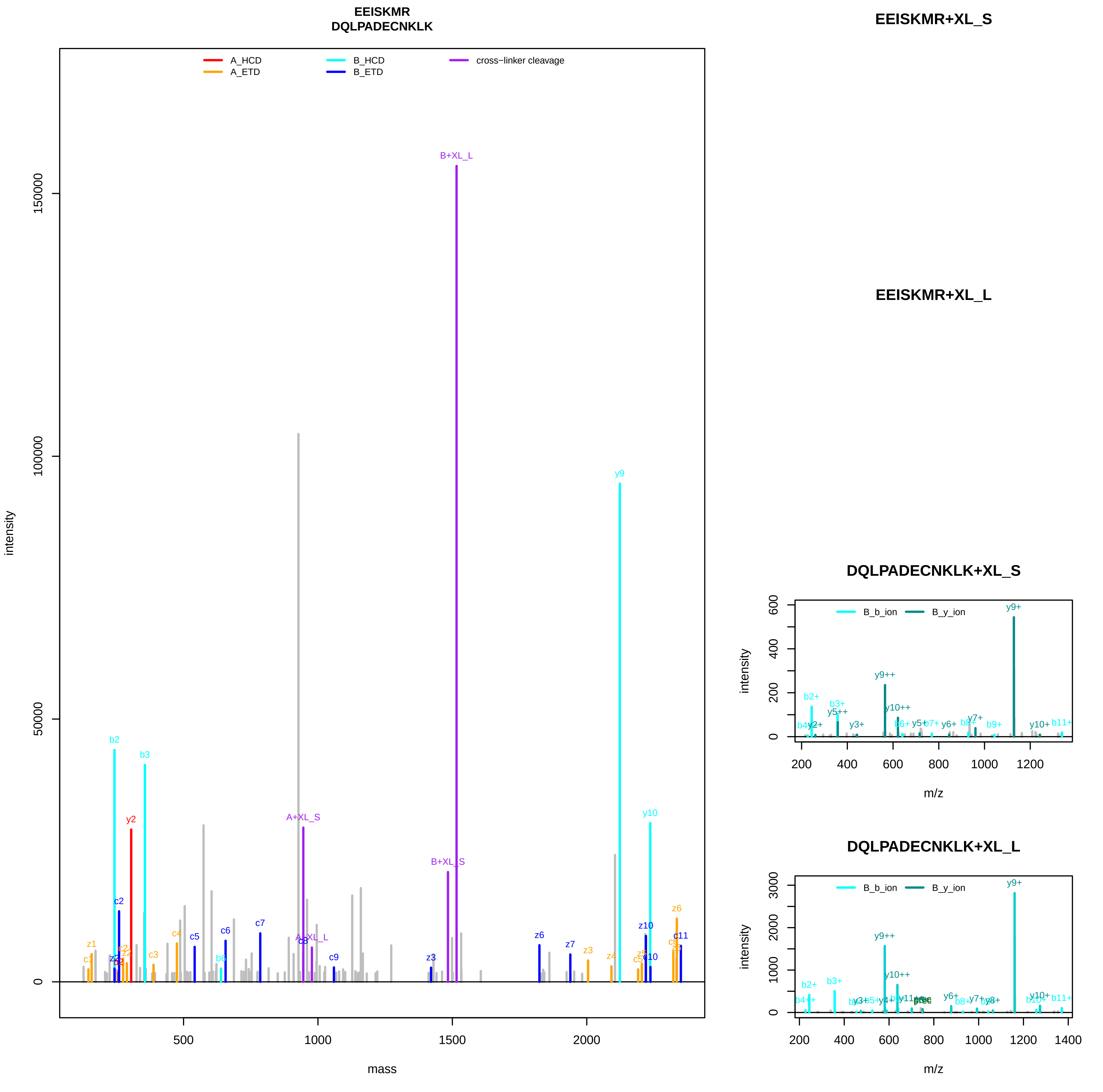
EEISKMR
DQLPADECNKLK
A_HCD
A_ETD
B_HCD
B_ETD
cross−linker cleavage
B+XL_L
y9
y10
b2
b3
y2
c2
c5
c6
c7
c9
z3
z6
z7
z3
z4
z10
c10
c11
z6
z1
c1
c3
c4
b6
A+XL_S
A+XL_L
B+XL_S
intensity
mass
0
50000
100000
150000
500
1000
1500
2000
EEISKMR+XL_S
EEISKMR+XL_L
DQLPADECNKLK+XL_S
B_b_ion
B_y_ion
y9+
y9++
y10++
b2+
b3+
y5++
y3+
y5+
y6+
y7+
b9+
y10+
b11+
intensity
m/z
0
200
400
600
200
400
600
800
1000
1200
DQLPADECNKLK+XL_L
B_b_ion
B_y_ion
y9+
y9++
y10++
b2+
b3+
y6+
y7+
y8+
y10+
b11+
intensity
m/z
0
1000
2000
3000
200
400
600
800
1000
1200
1400
